# Exploring Pet Owner Preferences in Order to Assess the Role of Cost and Quality of Life in Anti-Pruritic Treatment Plan Selection for Dog Owners

**DOI:** 10.3390/ani15040509

**Published:** 2025-02-11

**Authors:** Andrea Wright, Edwina Gildea, Michelle Greaves, Louise Longstaff, Robin Wyn, Nirav Nagda, Kristina DiPietrantonio, Ashley Enstone, Danielle Riley, David Bartram

**Affiliations:** 1 Zoetis Global Medical Affairs, 10 Sylvan Way, Parsippany, NJ 07054, USA; 2 Zoetis UK Ltd., First Floor, Birchwood Building, Springfield Drive, Leatherhead, Surrey KT22 7LP, UK; edwina.gildea@zoetis.com (E.G.);; 3 Adelphi Values PROVE, Adelphi Mill, Bollington, Cheshire SK10 5JB, UK; 4 Outcomes Research, Zoetis International Operations, Loughlinstown, D18 T3Y1 Dublin, Ireland

**Keywords:** canine pruritus, outcomes research, quality of life, treatment preference, willingness-to-pay

## Abstract

A web-based quantitative survey tested pet owners’ preferences and willingness-to-pay for canine anti-pruritus therapies, and for improvements in pruritus-related quality of life. This survey was designed using findings from recent research into quality of life in pruritus, interviews with pet owners and veterinarians, and clinical and cost data. The survey was completed by 251 dog owners in the United Kingdom. A total of 46–47% were willing to pay to improve individual concepts of pruritus-related quality of life, including comfort, scratching behavior, and appearance. Instituting treatment to manage pruritus and the investigation of pruritus causes were considered important actions; safety and effectiveness were the most important attributes of therapy. Comparing the administration, effectiveness, safety, and costs of (unbranded) therapy profiles, on average, 63% preferred hypothetical tablet or injectable therapies (with higher cost and improved safety) over corticosteroid treatment for acute pruritus (*p* < 0.05). Over 50% of respondents preferred the hypothetical therapies in all tested subgroups, and results were similar for chronic pruritus. This research highlighted that many pet owners are willing to pay to improve their pet’s quality of life, and to receive comparably effective, yet safer therapies for the management of acute canine pruritus, regardless of insurance status. Veterinarians should consider discussing and offering newly available therapies for acute and chronic pruritus with pet owners where medically appropriate.

## Simple Summary

Itch, known as pruritus, is known to reduce the quality of life of both pet dogs and their owners. However, dog owners’ opinions about treatments for canine pruritus are not well known. Using a web-based quantitative survey, we found that almost half of UK’s dog owners were willing to pay for more effective treatments against canine pruritus to improve their dog’s quality of life regardless their financial status. More than half of the dog owners that took part in the survey preferred safer and newer treatment options, and as such, veterinary clinics should discuss and offer newly available therapies for acute and chronic pruritus with pet owners where medically appropriate.

## 1. Introduction

Skin disease is among the most prevalent conditions among dogs presented to veterinarians [1], and pruritus, or itching, is a hallmark sign [2]. Pruritus may result from several factors, most commonly including (but not limited to) ectoparasitic infestation (such as by fleas or ticks), skin infection (by bacteria and/or yeast), and reactions to food or environmental allergens, and may manifest at the site of exposure or elsewhere on the body [3,4,5].

The behaviors associated with canine pruritus, including scratching, licking, biting, or chewing, and issues such as low energy and poor sleep, negatively affect the quality of life (QoL) of a dog and its owner [6,7], and may be a significant driver for the owner to seek treatment for their dog. Prior outcomes research has been conducted in this area, which has illustrated key concepts (such as happiness, playfulness, quality of sleep, and positive family interaction) that relate to the freedom to express normal patterns of behavior [8], and which can be harmed with canine pruritus, thereby reducing canine and pet owner QoL [7,9,10,11].

Multiple pharmacological options are available for the management of acute canine pruritus [12], including conventional corticosteroid therapies, and newer more targeted therapies such as oclacitinib and lokivetmab [13,14], which are associated with reduced side-effect burden [15,16,17]. However, pet owners’ perceptions of these varied therapy options are not known.

In light of the above, including the potential key influence of cost on treatment selection in this area, a quantitative study methodology (focusing on aspects determined through qualitative interview research) was applied to determine pet owners’ preferences for varied therapy characteristics and concepts of QoL in canine pruritus.

The discrete-choice experiment (DCE, or “conjoint analysis”) methodology is established in health preference research [18]. In this methodology, respondents consider profiles that are described in terms of one or more attributes, each of which is set at a defined level. Respondents are presented with choice sets including two or more profiles, from which they select their preferred options (thereby making trade-offs) [18]. DCE is a widely used methodology when exploring individuals willingness-to-pay (WTP) and preference for proposed products or concepts [19].

Similar quantitative studies have been conducted to measure preferences around companion animal breeding, acquisition, and feeding [20,21,22], as well as the conservation and farming of other animals [23,24,25]. However, research into preferences in companion animal health appears limited [26,27].

In order to clarify dog owners’ preferences for and WTP for anti-pruritus therapies in the United Kingdom, within a sample size consistent with prior DCE studies, a mixed-methods approach including an initial qualitative research stage, followed by a web-based quantitative survey, was conducted. This particularly related to the impact of pruritus on QoL. The aim of this research was to understand the relative importance of several concepts of dog and owner QoL relating to canine itch, and to estimate the pet owner’s willingness to pay for various anti-pruritic therapies in this area.

## 2. Materials and Methods

**Figure 1:**
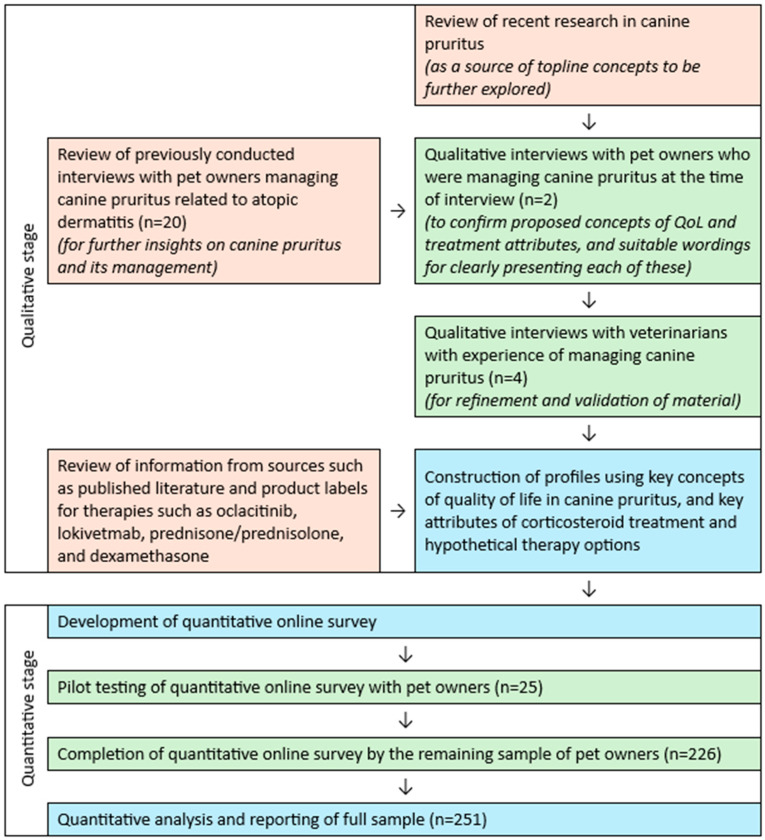
Flow chart of qualitative and quantitative research activities within this study.

A mixed-methods approach was undertaken, where qualitative findings were generated and gathered in order to inform the development of a quantitative survey (see Figure 1).

In short, the quantitative survey included trade-off questions between QoL concepts to reveal their relative importance, a conjoint analysis section involving the comparison of therapy profiles (with or without an associated cost) to test willingness to initiate treatment, and questions testing respondents’ level of agreement with statements on canine pruritus management (as described below).

All qualitative and quantitative data collection was undertaken in 2020.

*Ethics statement*

As this research was purely non-interventional in nature, ethics approval was not sought. All respondents provided expressed informed consent to participate after receiving information on study aims and procedures, confidentiality of personal data, and other aspects. All respondents received financial compensation according to fair market value for their participation in the survey.

*Qualitative interviews with pet owners and veterinarians*

Recent research into QoL in canine pruritus [9,10,11], insights from new qualitative interviews with pet owners and veterinarians, and information from sources such as product labels were first gathered during a qualitative stage, to allow for the later development of a quantitative survey.

Recent research in canine pruritus was used as a source of topline concepts to be further explored in the later stages of this study [9,10,11]. Concepts included the domains of canine QoL and pet owners’ QoL that are affected by pruritus, and pet owners’ satisfaction with treatment for pruritus [9,10,11]. Key concepts for inclusion within the quantitative survey were then determined through qualitative research in the United Kingdom.

Proposed concepts of QoL and treatment attributes, and suitable wordings for clearly presenting each of these, were confirmed through first-stage qualitative interviews with dog owners who were managing pruritus in their pet at the time of interview (*n* = 2). Dog owners initially were asked exploratory questions regarding their perceptions of canine pruritus and its management, before specific QoL concepts were presented and discussed (see Table 1).

Further insights were also captured from a review of previously conducted interviews with pet owners managing canine pruritus related to atopic dermatitis (*n* = 20), from which information applicable to pruritus and its management in general was extracted [11]. The analysis of key themes, concepts, and wording identified within these previous results was undertaken in order to determine any common issues experienced with the administration and effectiveness of anti-pruritic therapies in canines.

The first stage identified three major concepts of QoL impacted by pruritus: behavior (including fixation on scratching and other behaviors, which may interrupt play and sleep), appearance (including skin inflammation and damage), and comfort (including a pet’s agitation, distraction, or low mood). Three major attributes of anti-pruritus therapies were also identified: mode and frequency of administration, side-effect profile, and onset and duration of effect.

The material developed at the first stage was then refined and validated in second-stage qualitative interviews with veterinarians with experience of managing canine pruritus (*n* = 4). Veterinarians were initially asked exploratory questions relating to canine pruritus and its management, before being asked to react to the written material developed at the first stage (see Table 2). Changes made at this stage included emphasizing the signs of discomfort and restless nature of a dog affected by pruritus (and negative effect on the pet owner), the addition of further information on skin changes, and clarifications of descriptions of safety.

In order to describe realistic therapy profiles, information on product characteristics such as effectiveness, administration, and safety were collected.

Hypothetical product profiles informed by existing therapies, incorporating data from sources such as published research and product labels, were developed for therapies such as oclacitinib, lokivetmab, prednisone/prednisolone, and dexamethasone [15,16,28,29,30,31,32,33]. Realistic “out-of-pocket” costs were developed according to estimates provided by a practicing UK veterinarian and both starting and maintenance doses were incorporated (if relevant). These estimates reflected the costs that an individual would pay if purchasing the prescribed medication(s), per course or per month, incorporating standard costs such as drug product cost, retailer markup, and applicable taxes in the UK. For therapies that are dosed variably based on weight, a conservative estimate was used (27 kg), based on the weight of a medium-sized pet (e.g., adult Labrador).

Using the above qualitative and clinical information, profiles were constructed using key concepts of QoL in canine pruritus, and key attributes of corticosteroid treatment and hypothetical therapy options.

*Quantitative survey of pet owners*

The concepts validated in the qualitative research were incorporated into a quantitative survey to determine the relative importance of key concepts of QoL in canine pruritus, and to understand preferences between therapies for its management (see Supplementary Materials Figure S1 for overview).

Profiles on QoL (Table 3) specified dog behavior (including frequency of scratching and other disruptive activities), appearance (including color and condition of coat and skin), and comfort (including ability to rest, and overall demeanor). Each profile presented two or three of these concepts in untreated or treated states, in order to allow respondents to make trade-offs, and thereby reveal the relative importance of these concepts.

Trade-off questions on QoL profiles tested each possible combination of the three concepts, and also included an additional test question, where one profile described treated behavior and skin, whereas the other described untreated behavior and skin (with no trade-off); any respondent who answered the test question incorrectly was fully excluded from the final analysis.

Profiles on therapy options (Table 4) specified the mode and frequency of administration (including requirements for dose tapering, if relevant), the speed of onset and length of effectiveness, and major side-effects; the peak effectiveness of each therapy was considered equivalent, and therefore was not included. Profiles were therefore presented to pet owners with the assumption of comparable effectiveness across hypothetical products.

In addition, further information on long-term treatment (including side-effect burden and monitoring requirements) was added in profiles that tested preferences for long-term treatment regimens, thereby generating findings indicative of the preferences of owners of canines with chronic pruritus. As the length of treatment of chronic pruritus cannot be predicted while the condition is ongoing, an indefinite period of treatment was described within these profiles. It should be noted that pet owner preferences for QoL attributes (such as scratching behavior, comfort, and appearance, and product attributes such as effectiveness, safety, and administration) were tested separately within this study; therefore, findings on QoL and product attributes may be considered independently of each other.

Further tasks included a conjoint analysis section involving therapy profiles presented (with or without an associated cost) to test willingness to initiate that treatment, and questions testing respondents’ level of agreement (by rating scale) with general statements around canine pruritus and its management.

Tasks including therapy profiles included the therapies specified in Table 4. In each case, tablet A or injection B were compared to the existing standard of care, which was corticosteroid treatment. Although all included therapy profiles were based on characteristics and clinical data of therapies for canine pruritus, these therapies were not named or branded. Respondents were also explicitly instructed to assume that all other factors (e.g., availability, recommendation status) were completely equal between each profile, and that their decision should be based only on the provided information.

Prior to full launch, the survey underwent pilot testing by 25 respondents, who were recruited using criteria that were identical to those used for the full launch. Pilot testing data were examined to determine if the survey was easily understood by pet owners, and the survey design and programming were amended where appropriate.

### 2.1. Quantitative Survey Respondent Screening Process and Criteria

Respondents were recruited from pre-existing panels of research participants maintained by a third-party research agency (Qualtrics). Potential respondents were contacted via email to participate in the survey.

No prior assumption was made regarding the direction or magnitude of pet owners’ preferences prior to conducting this survey. In addition, a range of question types were included to address several co-primary outcomes. Therefore, no formal power calculation was conducted. A target sample size of 250 complete responses was therefore sought, based on precedents from the published literature [34,35,36,37,38,39,40,41,42,43,44,45].

Eligible respondents were adult (age ≥ 18) residents in the United Kingdom, who owned at least one pet dog, but did not own more than five dogs and/or cats (in total) at the time of the survey.

Eligible respondents were the individual solely or jointly responsible for decisions regarding veterinary products for their dog(s) and reported adequate literacy in terms of veterinary information. Respondents reported their reliance on help when reading veterinary materials, confidence filling out veterinary forms, and frequency of problems when reading veterinary materials; scores from 1 to 5 were assigned to each answer. Adequate literacy was defined as a score of 4 or 5 on each question, where 5 represented lowest reliance on help, highest confidence, and lowest frequency of problems, respectively.

Additionally, quotas were applied to ensure that a representative sample of respondents were captured from various regions (or groups of regions) across the United Kingdom, according to the population of each [46]. Specifically, 13–32% of respondents were required to be a resident of each of the following regions: London; South and East England (excluding London); the Midlands; the North (including Yorkshire and the Humber); and the other home nations (Scotland, Wales, and Northern Ireland).

Further demographic information and information on pet ownership characteristics (such as numbers of pet dogs and cats) were also collected to aid in the interpretation of results and identify key subgroups of interest.

### 2.2. Quantitative Survey Data Analysis

Survey results were collated and underwent quality checks prior to analysis (including removal of highly unrealistic suggested WTP values, or incoherent free-text responses). Respondents were also excluded according to the criteria reported in the Survey respondent subsection, if applicable.

Demographic data were analyzed descriptively. Differences in preference between treatments were examined using separate chi-squared tests for each question (with “treatment profile” as the predictor variable, and “proportion of respondents preferring” as the outcome variable), and differences in suggested WTP values were assessed using a separate one-way Analysis of Variance (ANOVA) for each question (with “treatment profile” or “improvement in concept of QoL” as the predictor variable, and “respondents’ willingness to pay” as the outcome variable). Statistical significance was assessed versus a threshold of *p* < 0.05, and *p* values were not adjusted for multiple testing, as only a single test was conducted and reported on each question. Subgroup analyses based on demographic variables, such as the presence or absence of pet health insurance, were also conducted using chi-squared testing. In the survey, cost data were presented and collected in GBP only, but here are also presented in USD and EUR to aid interpretation (GBP 1 = approximately US 1.26, EUR 1.17 on 04 September 2023).

In addition, regression analyses were conducted to examine the influence of subgroup characteristics on the likelihood of being willing to pay an additional cost to manage specific concepts of QoL related to itch, or for hypothetical treatment profiles. Specifically, a probabilistic ordinary least squares regression model was developed in R, examining key patient characteristics.

## 3. Results

A total of 251 dog owners from the United Kingdom provided complete and analyzable responses to the quantitative survey, fulfilling the target sample size.

### 3.1. Pet Owner Quantitative Survey Respondent Characteristics

The sample recruited was geographically representative of the UK population and covered a wide range of age groups and sociodemographic backgrounds (see Table 5).

Information on respondents’ pets was also collected (see Table 5). The majority of respondents had one household dog (72%) and no cats (79%). The majority of respondents had health insurance for their dog (68%), and approximately half of respondents (47%) had experience of their current or past pet being affected by canine pruritus.

### 3.2. Pet Owner Quantitative Survey Responses: Perceptions of Canine Pruritus Management

**Figure 2:**
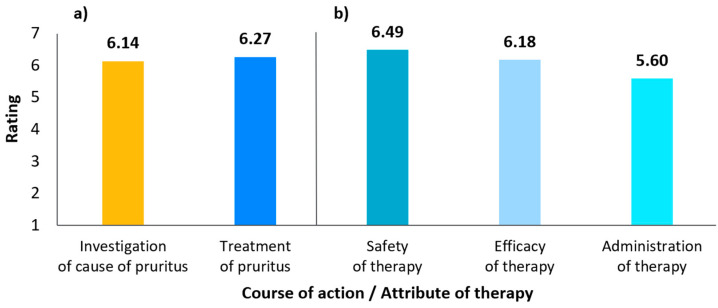
(**a**) Relative importance of actions taken to manage pruritus (**b**) Relative importance of therapy attributes (mean values; 1 being not at all important and 7 being very important) in a quantitative survey of pet owners.

When asked to rate the importance of actions taken to manage canine pruritus (with 1 being “not at all important” and 7 being “very important”), the management of pruritus and investigation of the cause of pruritus were both considered important (mean rating 6.3 and 6.1 out of 7, median rating of 7 and 7 out of 7, respectively; see Figure 2a). On the same scale, safety was rated as the most important attribute of a pruritus therapy, followed by effectiveness and then administration (mean rating 6.5, 6.2, and 5.6 out of 7, median rating 7, 7, and 6 out of 7, respectively; Figure 2b).

### 3.3. Pet Owner Quantitative Survey Responses: Preferences for Improvements in QoL Related to Canine Pruritus

When offered separate hypothetical courses of treatment (costing GBP 28 each; USD 38, EUR 31) that each improved only two of three concepts of QoL, 46–47% of respondents were willing to pay extra to improve the remaining concept (for each of behavior, appearance, and comfort). Respondents were willing to pay a mean extra of GBP 24 (USD 33, EUR 27) per course of treatment, and there was no significant difference between concepts for the proportion of respondents willing to pay more (by chi-squared test) or mean extra WTP (by one-way ANOVA).

When asked to directly trade-off these concepts of QoL (by selecting between profiles where one concept was improved whereas another was still badly affected, and vice versa), improving their pet’s comfort was most preferred by respondents: comfort was preferred by the majority in both comparisons (75–80%), and the opposite was true for appearance (20–38%), while behavior was chosen less often than comfort (25%), but more often than appearance (62%; Table 6).

### 3.4. Pet Owner Quantitative Survey Responses: Preferences for Canine Pruritus Therapy Profiles

When offered an (unlabeled) therapy profile representing corticosteroid treatment with dexamethasone and prednisone/prednisolone (costing GBP 28 per course; USD 38, EUR 31), 50% and 49% of respondents were willing to pay extra to instead manage acute itch with tablet A or injection B, respectively. Respondents were willing to pay a mean extra of GBP 21 or GBP 22 per course of treatment, respectively (USD 28 or USD 30; EUR 24 or EUR 25), and there was no significant difference between tablet A and injection B for the proportion of respondents willing to pay more (by chi-squared testing) or mean extra WTP (by ANOVA).

**Figure 3:**
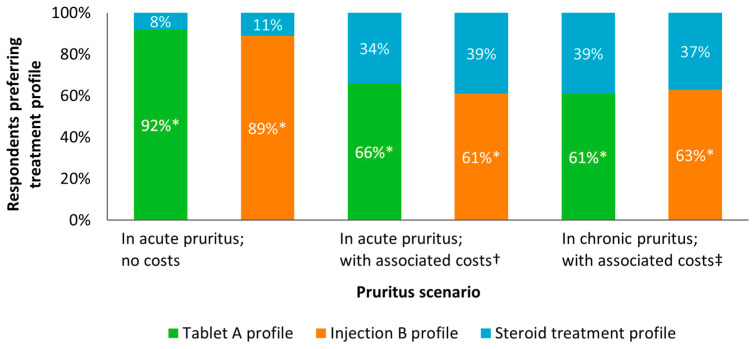
Preferences for canine pruritus therapy profiles, in direct comparisons, in a quantitative survey of pet owners. * Significantly preferred with *p* < 0.005 by chi-squared testing. † Costs in acute pruritus (per course): corticosteroid treatment (dexamethasone and prednisone/prednisolone) was GBP 28 (USD 38, EUR 31); tablet A was GBP 93.50 (USD 127, EUR 105); injection B was GBP 110 (USD 149, EUR 123). ‡ Costs in chronic pruritus (per month): corticosteroid treatment (dexamethasone and prednisone/prednisolone) was GBP 28 (USD 38, EUR 31); tablet A was GBP 100 (USD 135, EUR 112); injection B was GBP 110 (USD 149, EUR 123). Chi-squared testing results in detail: In acute pruritus, no costs: 92% preferred tablet A, chi-squared (1 degree of freedom; *n* = 251) = 174.03; *p* < 0.005. In acute pruritus, no costs: 89% preferred injection B, chi-squared (1 degree of freedom; *n* = 251) = 151.49; *p* < 0.005. In acute pruritus, with associated costs: 66% preferred tablet A, chi-squared (1 degree of freedom; *n* = 251) = 24.86; *p* < 0.005. In acute pruritus, with associated costs: 61% preferred injection B, chi-squared (1 degree of freedom; *n* = 251) = 12.94; *p* < 0.005. In chronic pruritus, with associated costs: 61% preferred tablet A, chi-squared (1 degree of freedom; *n* = 251) = 11.19; *p* < 0.005. In chronic pruritus, with associated costs: 63% preferred injection B, chi-squared (1 degree of freedom; *n* = 251) = 16.83; *p* < 0.005.

When asked to directly select between therapy profiles representing the administration, effectiveness, and safety of corticosteroid treatment and the hypothetical therapy profiles, with or without associated costs, and in acute or chronic pruritus scenarios, the hypothetical therapy profiles were always preferred by significantly more respondents (*p* < 0.005 in all cases, by chi-squared testing; Figure 3).

When asked to directly select between therapy profiles representing corticosteroid treatment and the hypothetical therapy profiles without associated costs in acute pruritus, the preference for tablet A or injection B was high: 92% and 89% selected these, respectively (both *p* < 0.005 over corticosteroid treatment, by chi-squared testing).

When selecting between therapy profiles representing corticosteroid treatment and the hypothetical therapy profiles, with associated costs (GBP 28 versus GBP 93.50 or GBP 110, respectively; USD 38 versus USD 127 or USD 149; EUR 31 versus EUR 105 or EUR 123), the preference for tablet A or injection B over corticosteroid treatment was still significant: 66% and 61%, respectively (both *p* < 0.05 by chi-squared testing).

When selecting between the same therapy profiles in chronic pruritus (with chronic-specific safety information and costs of GBP 28 versus GBP 100 or GBP 110; USD 38 versus USD 135 or USD 149; EUR 31 versus EUR 112 or EUR 123), the preference for tablet A or injection B over corticosteroid treatment was still significant: 61% and 63%, respectively (both *p* < 0.05 by chi-squared testing).

In all of the above scenarios, the proportion of respondents preferring both hypothetical therapy profiles was not lower than 50% in any tested subgroup defined by gender (male; female), age (18 to 39; 40 to 59; 60+ years), household income (below GBP 20k; GBP 20k to GBP 40k; GBP 40k+), geographical location (London and South; Midlands; North and other), or the presence of health insurance for dog (yes; no) (Supplementary Materials Table S1).

In regression analyses, undergraduate level of education (versus a secondary school level of education) was significantly associated with willingness to pay an extra cost to resolve behavior (+0.191; *p* < 0.05) and appearance related to itch (+0.187; *p* < 0.05). Holding pet insurance (versus not holding pet insurance) was also significantly associated with willingness to pay an additional cost to use tablet A rather than corticosteroid treatment in acute itch (+0.150; *p* < 0.05). The full results of regression analyses are presented in Supplementary Materials Table S2.

The majority of respondents appeared comfortable with allowing injection B to be administered by a veterinarian nurse, rather than a veterinarian: respondents were significantly more likely (65%; *p* < 0.005 by chi-squared testing; chi-squared (1 degree of freedom; *n* = 251) = 23.62)) to prefer veterinarian nurse administration (GBP 102.50 total cost; USD 138, EUR 115) over veterinarian administration (GBP 110 total cost; USD 149, EUR 123).

## 4. Discussion

This quantitative survey of pet owners in the United Kingdom demonstrated that 46–47% are willing to pay to improve individual concepts of pruritus-related QoL in terms of comfort, scratching behavior, and skin appearance. When directly compared, comfort is the most preferred concept in which to see improvements, followed by scratching behavior, and lastly skin appearance.

Hypothetical therapy profiles for pruritus appear to be preferred over corticosteroid treatment, in all scenarios. When considering treatment administration, effectiveness, and safety, approximately nine out of ten pet owners prefer hypothetical therapy profiles over corticosteroid treatment in acute pruritus. When considering treatment cost in addition to other factors, approximately six out of ten pet owners prefer hypothetical therapy profiles over corticosteroid treatment in acute pruritus. A similar level of preference is seen in chronic pruritus. The majority of pet owners preferred newer therapies regardless of income or pet insurance status, suggesting a broad willingness to pay an additional out-of-pocket cost for this option among pet owners in the UK. However, further research should be conducted to explore the direct impact demographic variables may have on pet owner preferences.

This study is notable as one of the few to have applied a DCE methodology and gathered WTP data in companion animal health [26,27]. The application of this trusted methodology within animal health has provided insight into key drivers in pet owner decision making. Additionally, despite this research forming one of the few WTP studies conducted in companion animal health, the sample size for this research (*n* = 251 quantitative survey responses) is generally consistent with those of other discrete-choice studies in animal and human health, which typically analyze 100 to 500 responses [34,35,36,37,38,39,40,41,42,43,44,45].

The limitations of this study are reflective of the quantitative methodology that was used. As the survey was administered prospectively to pet owners who were willing to participate, there was a risk that the sample could be biased towards demographic groups who are more willing to participate in research (known as participation bias). In addition, there was a risk that participating pet owners could be systematically less likely or more likely to be willing to pay an increased price for treatment, relative to the wider population of pet owners in the UK. However, demographic data indicate that this sample appears broadly representative of the wider UK population [47].

Also, as this research surveyed pet owners regarding their willingness to pay an additional cost to improve outcomes for their pet, these findings may have been subject to “social desirability bias”, where respondents consciously or unconsciously alter their answers in order to appeal to observers. However, the anonymous nature of data collection and presentation may have reduced this risk.

Previous experience with pruritus was not a strict requirement, as we were interested in capturing the opinions of pet owners who may be asked to select between canine pruritus therapies at a later date. As such, 53% of survey respondents had no experience of managing canine pruritus, and therefore were reliant on the provided descriptions and profiles to inform their answers. However, as the incidence of canine pruritus is high, it is likely of interest to understand the preferences of pet owners seeking treatment for “first-time” acute canine pruritus.

The concise profiles of canine pruritus and related therapies used in the survey may not describe the full experiences of affected pets and their owners. However, profiles were informed by prior qualitative research [9,10,11] and clinical information [15,16,28,29,30,31,32,33], and were further validated by pet owners and veterinarians prior to the quantitative stage. The use of concise profiles also allowed the survey to collect preference data on both QoL and the management of pruritus.

Therapy profiles were designed according to the clinical data available at the time of the study (mid-2020); therefore, these survey findings should be interpreted in the context of available information in the future. New data on long-term use of newer therapies in canine pruritus (or further data on the long-term safety of treatment with corticosteroids) may reinforce or contradict the validity of the profiles presented here. In addition, readers should be aware that new treatment options are likely to become available (e.g., ilunocitinib, which was not an approved therapeutic option at the time this research was conducted) [48]. Also, as this study focused on binary outcomes (including pet owners’ likelihood of being willing to pay an additional cost, or pet owners’ likelihood of selecting a treatment profile versus corticosteroids), these data may not easily be extrapolated to other scenarios where treatment prices are different.

Costs within therapy profiles were not adjusted by region or socioeconomic status of respondents. In practice, out-of-pocket cost may vary due to consultation or administration fees, transport cost, or other factors. Subgroup analyses of results by region and socioeconomic status are presented, to place these findings in context. However, descriptive, and univariate statistical analyses may not be able to fully elucidate the potential interactions between these characteristics. Future research could focus on collecting data relating to these characteristics, and employ multivariate statistical methods, in order to understand how these factors may interact with each other and influence pet owners’ preferences.

It should also be acknowledged that the level of comfort of a pet is only known subjectively by the pet owner through their experience and observations, rather than being an objectively measured concept. As such, this study focused on presenting potential improvements in observable aspects of pets’ conditions (including behavior, appearance, and level of comfort) as perceived by the pet owner.

In addition, as the quantitative survey was administered during the COVID-19 pandemic, pet owners’ perceptions of activities such as veterinarian visits may have been altered in a way that is difficult to control for.

## 5. Conclusions

In conclusion, this research demonstrates that many pet owners are willing to pay to improve their and their pet’s QoL, and to receive more tolerable therapies in acute canine pruritus. A total of 46–47% were willing to pay to improve individual concepts of pruritus-related quality of life, including comfort, scratching behavior, and appearance. Comparing the administration, effectiveness, safety, and costs of (unbranded) therapy profiles, on average, 63% preferred newer tablet or injectable therapies (with higher cost and improved safety) over corticosteroid treatment for acute pruritus. Veterinarians should therefore consider discussing and offering newly available therapies for acute and chronic pruritus with pet owners where medically appropriate. Examining pet owners’ willingness to pay from the perspective of veterinarians and exploring the interaction between QoL changes and product attributes could be topics of interest for future research.

## Figures and Tables

**Table 1:** Key pruritus-related signs and behaviors, and pruritus therapy characteristics, for inclusion, as per findings from initial qualitative interviews with pet owners.

Key Pruritus-Related Signs and Behaviors Identified in Pet Owner Interviews	Key Pruritus Therapy Characteristics Identified in Pet Owner Interviews
Important signs related to physical appearance included red and inflamed skin and broken skin (with potential to lead to infection).	Side-effect profile, balanced versus the benefits of treatment.
Important behaviors included constant fixation on itching, biting, or licking; interruption of play due to itching; interruption of sleep due to itching.	Ease and frequency of administration.
Important factors related to comfort/demeanor included restlessness or agitation; distraction or reduced energy; low or reserved mood.	Onset and duration of treatment effect.

**Table 2:** List of changes to health state descriptions and therapy profiles made as a result of qualitative veterinarian interviews.

Changes to Health State Descriptions Made as a Result of Qualitative Veterinarian Interviews:
The restless nature of a dog affected by pruritus was emphasized.
Additional information was added about skin changes, including discoloration and smell.
General constant signs of discomfort (that have a negative effect on the pet owner) were emphasized.
Changes to therapy profiles made as a result of qualitative veterinarian interviews:
The characteristics of each tablet (corticosteroid and tablet A) were mentioned. ^a^
The requirement for a loading dose of tablet A was mentioned. ^a^
The dose tapering requirements associated with prednisolone were mentioned.
The side-effects associated with prednisolone were expanded upon.
The comparison of side-effects to placebo was clarified for easier understanding.
Chronic pruritus therapy profiles were created, with information on long-term side-effects and monitoring requirements.

^a^ Tablet A was a hypothetical and unbranded product profile based on the characteristics of oclacitinib.

**Table 3:** Key concepts of quality of life in canine pruritus displayed within the survey of pet owners.

Full Descriptions of Concepts, Presented as Introductory Text	
[Behavior—untreated] Your dog is fixated with scratching and other behaviors such as licking or biting, and often stops interacting with you due to becoming distracted. As your dog struggles to settle down, your sleep is often interrupted, along with your family activities.	[Behavior—treated] Your dog becomes much less fixated with scratching and related behaviors and interacts much more without becoming distracted. As your dog is able to settle down more easily, interruptions to your sleep or family activities are greatly reduced.
[Appearance—untreated] Your dog has very inflamed skin in the affected area, which has turned red and may become darker over time. The skin may become flaky or spotty and may smell. There is a risk that the skin will be broken, and an increased risk of infection.	[Appearance—treated] The inflammation of your dog’s skin is reduced, and the color steadily returns to normal. Any flakes, spots, or smell are now reduced. The risk of broken skin is now reduced, and the risk of infection is decreased.
[Comfort—untreated] Overall, your dog appears unhappy and uncomfortable, and often seems agitated. As your dog often shows signs of discomfort, you are constantly aware that they are suffering.	[Comfort—treated] Overall, your dog appears much happier and more comfortable, and now only rarely becomes agitated. As your dog seems more comfortable, you are less worried about their condition.
**Concise Descriptions of Concepts, Presented Within Trade-Off Tasks**	
[Behavior—untreated] Your dog… …is obsessed and distracted by scratching.	[Behavior—treated] Your dog… …is much less obsessed and distracted by scratching.
[Appearance—untreated] Your dog… …has red and damaged skin.	[Appearance—treated] Your dog… …has much more normal looking skin.
[Comfort—untreated] Your dog… …seems unhappy and uncomfortable.	[Comfort—treated] Your dog… …seems much happier and more comfortable.

**Table 4:** Key attributes of canine anti-pruritus therapies displayed within the survey of pet owners.

Information Included in Acute Pruritus Therapy Profiles		
Prednisone/prednisolone (tablet) + dexamethasone (injection)	Tablet A	Injection B
An injection, administered by your veterinarian once every month, and a small white tablet, administered once daily, which can be hidden in food.	A small white tablet, administered once daily, which can be hidden in food. For the first two weeks, you give this tablet twice daily.	An injection administered by your veterinarian once every month.
After the first week, you give this tablet once every other day. You must give this reduced dose for at least two weeks before stopping the treatment.	You can stop using this treatment at any time (with no need to reduce the dose over time before stopping).	You can stop using this treatment at any time (with no need to reduce the dose over time before stopping).
This treatment begins to take effect after 4 h and is fully effective after 24 h.	This treatment begins to take effect after 4 h and is fully effective after 24 h.	This treatment begins to take effect after 8 h and is fully effective after 48 h. An injection is effective for one month.
While on treatment, 30% of dogs show excessive urination, and 24% of dogs eat excessively. Dogs receiving this treatment are significantly more likely to show signs of anxiety, such as nervousness, barking, and aggressive reactions.	The side-effects of this treatment are similar to that of a placebo tablet. (A placebo tablet doesn’t contain any medicine.)	The side-effects of this treatment are similar to that of a placebo injection. (A placebo injection doesn’t contain any medicine.)
**Additional Information Added to Chronic Pruritus Therapy Profiles**		
Prednisone/prednisolone (tablet) + dexamethasone (injection)	Tablet A ^a^	Injection B ^b^
Dogs receiving this treatment over a long period have a significant risk of developing diabetes, and a hormone condition: the symptoms are obesity, thinning of skin and fur, and a weakened immune system.	The side-effects of this treatment are similar to that of a placebo tablet. (A placebo tablet doesn’t contain any medicine.)	The side-effects of this treatment are similar to that of a placebo injection. (A placebo injection doesn’t contain any medicine.)
It is recommended that your dog receives a blood test every six months while receiving this treatment.	It is recommended that your dog receives a blood test every twelve months while receiving this treatment.	Your dog does not need any additional blood test while receiving this treatment.

^a^ Tablet A was a hypothetical and unbranded product profile based on the characteristics of oclacitinib. ^b^ Injection B was a hypothetical and unbranded product profile based on the characteristics of lokivetmab.

**Table 5:** Demographic characteristics of respondents and their pets from a quantitative survey of pet owners.

Respondent Characteristic	% of *n* = 251
Gender	
Male	34%
Female	66%
Age category	
18–30	14%
31–40	22%
41–50	23%
51–60	24%
60+	18%
Household income category (£)	
<20,000	29%
20–40,000	37%
40–60,000	21%
60–80,00	7%
>80,000	6%
Geographical location	
London	14%
North East/North West/Yorkshire and Humberside	22%
Scotland/Wales/Northern Ireland	14%
South West/South East/East	33%
West Midlands/East Midlands	16%
**Characteristics of Respondents’ Pets**	**% of *n* = 251, or *n***
Number of household dogs (all respondents had at least 1)	
1	72%
2	26%
3	2%
Number of household cats	
0	79%
1	12%
2	7%
3	1%
4	1%
Health insurance for pet dogs	
Pet owners with health insurance for dog	68%
Pet owners without health insurance for dog	32%
Canine pruritus diagnosis	
Respondent with current experience	34%
Respondent with current or past experience	47%
Respondent with no experience	53%
Treatments received ^a^ by current or previously owned dog with pruritus	
Antihistamine tablets (e.g., trimeprazine)	53 respondents
Corticosteroids given as a tablet (e.g., prednisolone or prednisone)	43 respondents
Corticosteroids injection (e.g., dexamethasone)	15 respondents
Oclacitinib tablets	9 respondents
Other (e.g., ointment, shampoo)	7 respondents
Lokivetmab injection	1 respondent

^a^ Respondents could report use of more than one therapy.

**Table 6:** Proportion of respondents preferring to improve concepts of canine pruritus-related quality of life, in a quantitative survey of pet owners.

	…to Behavior	…to Appearance	…to Comfort
Comparing Behavior…	--	62% preferred improving behavior over appearance	25% preferred improving behavior over comfort
Comparing Appearance…	38% preferred improving appearance over behavior	--	20% preferred improving appearance over comfort
Comparing Comfort…	75% preferred improving comfort over behavior	80% preferred improving comfort over appearance	--

## Data Availability

The raw data supporting the conclusions of this article will be made available by the authors on request.

## Supplementary Materials

The following supporting information can be downloaded at: https://www.mdpi.com/article/10.3390/ani15040509/s1, Figure S1: Structure and content of quantitative survey, in detail; Table S1: Proportion of respondents who chose tablet A or injection B over steroid treatment; Table S2: Effect of subgroup on likelihood of being willing to pay an additional cost.
