# Supplementary material for: Exploring Pet Owner Preferences in Order to Assess the Role of Cost and Quality of Life in Anti-Pruritic Treatment Plan Selection for Dog Owners
Source: Animals (Basel). 2025 Feb 11;15(4):509. doi: 10.3390/ani15040509 (PMC11851607; doi:10.3390/ani15040509)
Supplement: Supplementary file 1 [file animals-15-00509-s001.zip › animals-3330978-supplementary.pdf]

Supplementary Figure S1. Structure and content of quantitative survey, in detail

Screening questions, further questions on demographic and pet details, and introductory information	
↓	
×2 questions on perceptions of canine pruritus management and therapy	— testing relative importance of pruritus investigation and treatment, and therapy attributes
↓	
×3 direct trade-off questions on improving domains of quality of life	— testing which domains respondents prefer to improve
×1 test trade-off question with a clearly inferior answer	— confirming respondents' understanding of the trade-offs
↓	
×3 questions on willingness to pay to improve domains of quality of life with therapy	— testing which domains respondents would pay to improve, and how much
↓	
×2 questions on willingness to pay to receive newer therapies (tablet A and injection B) rather than steroid treatment, in acute pruritus	— testing respondents' willingness to pay to receive newer therapies for pruritus
↓	
×6 questions on preference between steroids and newer therapies (tablet A and injection B):	— testing preferences between therapies:
in acute pruritus, with no associated cost	*in terms of their administration and safety*
in acute pruritus, with cost information	*when cost is a consideration*
in chronic pruritus, with cost information	*in a chronic scenario, with chronic-specific side-effects and monitoring requirements*
↓	
×1 question on preference between veterinarian and veterinarian nurse administration of injection B	— testing if respondents are comfortable with injectable therapies delivered by veterinarian nurses
↓	
Conclusion and contact information	

Supplementary Table S1. Proportion of respondents who chose tablet A or injection B over steroid treatment

Subgroup	In acute pruritus, no associated costs		In acute pruritus, with cost information		In chronic pruritus, with cost information	
	Respondents choosing tablet A over steroid treatment	Respondents choosing injection B over steroid treatment	Respondents choosing tablet A over steroid treatment	Respondents choosing injection B over steroid treatment	Respondents choosing tablet A over steroid treatment	Respondents choosing injection B over steroid treatment
Full sample	*89%*	*89%*	*66%*	*61%*	*61%*	*63%*
Gender						
Male	92%	87%	67%	58%	58%	58%
Female	92%	90%	65%	63%	62%	66%
Age						
18 to 39	91%	94%	76%	68%	65%	71%
40 to 59	91%	91%	65%	62%	64%	64%
60+	98%	87%	64%	58%	58%	60%
Household income						
<£20k	92%	86%	60%	55%	52%	58%
£20–40k	91%	90%	68%	65%	65%	64%
£40k+	92%	90%	67%	63%	63%	66%
Health insurance for dog						
Yes	92%	91%	69%	63%	65%	66%
No	90%	85%	59%	58%	52%	57%
Location						
London and South	93%	94%	67%	64%	63%	64%
Midlands	88%	80%	61%	61%	54%	61%
North and other	91%	86%	66%	58%	61%	62%

Supplementary Table S2. Effect of subgroup on likelihood of being willing to pay an additional cost

	Level of association between characteristic and willingness to pay an additional cost to treat specific domains of canine QoL related to itch, or for newer treatment options:				
	Improve behavior relating to itch:	Improve appearance relating to itch:	Improve comfort relating to itch:	Use Tablet A, rather than steroid treatment (acute scenario):	Use injection B, rather than steroid treatment (acute scenario):
Age: 18 to 30	0.098	0.135	0.198	0.084	0.067
	(0.154)	(0.153)	(0.153)	(0.156)	(0.155)
Age: 31 to 40	0.183	0.202	0.1	0.123	0.179
	(0.134)	(0.133)	(0.133)	(0.135)	(0.134)
Age: 41 to 50	0.177	0.107	0.083	0.228*	0.206
	(0.129)	(0.128)	(0.128)	(0.129)	(0.129)
Age: 51 to 60	0.093	0.092	-0.02	0.191	0.155
	(0.124)	(0.123)	(0.122)	(0.124)	(0.123)
Region: London	0.135	0.062	0.139	0.048	-0.062
	(0.121)	(0.12)	(0.119)	(0.121)	(0.12)
Region: North East/ North West/Yorkshire and the Humber	0.041	0.101	0.006	0.084	0.049
	(0.106)	(0.106)	(0.106)	(0.107)	(0.106)
Region: South West/ South East/East of England	-0.1	-0.111	-0.069	-0.032	-0.104
	(0.098)	(0.098)	(0.097)	(0.098)	(0.098)
Region: Scotland/Wales/ Northern Ireland	0.095	0.017	0.03	0.0003	0.082
	(0.118)	(0.118)	(0.117)	(0.119)	(0.118)
Number of dogs in household	-0.038	-0.021	0.031	-0.023	-0.042
	(0.066)	(0.066)	(0.066)	(0.066)	(0.066)
Number of cats in household	-0.069	-0.074	-0.018	-0.04	-0.058
	(0.046)	(0.046)	(0.045)	(0.046)	(0.046)
Gender: male	-0.059	0.013	-0.045	0.065	-0.009
	(0.071)	(0.07)	(0.07)	(0.071)	(0.071)
Level of education: diploma	0.06	-0.005	0.002	-0.105	-0.08
	(0.081)	(0.08)	(0.08)	(0.081)	(0.081)
Level of education: undergraduate	0.191**	0.187**	0.149*	0.07	0.067
	(0.088)	(0.087)	(0.087)	(0.088)	(0.088)
Level of education: postgraduate	NA	NA	NA	NA	NA
	NA	NA	NA	NA	NA
Level of education: doctorate	NA	NA	NA	NA	NA
	NA	NA	NA	NA	NA
	-0.035	-0.039	-0.012	-0.148	-0.139

	Level of association between characteristic and willingness to pay an additional cost to treat specific domains of canine QoL related to itch, or for newer treatment options:				
	Improve behavior relating to itch:	Improve appearance relating to itch:	Improve comfort relating to itch:	Use Tablet A, rather than steroid treatment (acute scenario):	Use injection B, rather than steroid treatment (acute scenario):
Employment status: full-time	(0.096)	(0.096)	(0.095)	(0.096)	(0.096)
Employment status: part-time	-0.076	-0.025	-0.061	-0.17	-0.111
	(0.104)	(0.104)	(0.103)	(0.105)	(0.104)
Employment status: student	0.046	0.222	-0.053	-0.117	0.236
	(0.24)	(0.238)	(0.238)	(0.242)	(0.24)
Employment status: retired	0.137	0.092	0.153	0.083	0.129
	(0.142)	(0.141)	(0.14)	(0.142)	(0.141)
Income: over £80k	0.051	-0.042	0.173	0.162	0.187
	(0.154)	(0.153)	(0.153)	(0.155)	(0.154)
Income: £60k to £80k	0.15	0.148	0.202	0.249*	0.182
	(0.145)	(0.144)	(0.143)	(0.145)	(0.144)
Income: £40k to £60k	-0.02	-0.165	-0.127	0.039	0.008
	(0.1)	(0.101)	(0.099)	(0.1)	(0.1)
Income: £20k to £40k	-0.027	-0.088	-0.105	0.036	0.065
	(0.088)	(0.087)	(0.087)	(0.088)	(0.088)
Insurance status: has pet insurance	0.051	0.041	0.102	0.150**	0.121*
	(0.073)	(0.073)	(0.073)	(0.073)	(0.073)
Experience of canine itch: has experienced canine itch, now or in the past	0.025	0.012	0.016	0.034	0.052
	(0.07)	(0.07)	(0.069)	(0.07)	(0.07)
Constant	*0.214*	*0.253*	*0.185*	*0.146*	*0.207*
	(0.194)	*(0.193)*	*(0.192)*	*(0.195)*	*(0.193)*

Note: A positive constant represents that the baseline individual would be more likely to be willing to pay than not; other coefficients represent the influence of each subgroup characteristic on likelihood of being willing to pay (with associated standard errors).

*p<0.1; **p<0.05; ***p<0.01.

Note: p<0.05 was considered to indicate statistical significance.
